# Isolation and Characterization of Six *Vibrio parahaemolyticus* Lytic Bacteriophages From Seafood Samples

**DOI:** 10.3389/fmicb.2021.616548

**Published:** 2021-03-10

**Authors:** Chia Wanq Tan, Yaya Rukayadi, Hanan Hasan, Noor-Azira Abdul-Mutalib, Nuzul Noorahya Jambari, Hirofumi Hara, Tze Young Thung, Epeng Lee, Son Radu

**Affiliations:** ^1^Department of Food Science, Faculty of Food Science and Technology, Universiti Putra Malaysia, UPM Serdang, Selangor, Malaysia; ^2^Food Safety and Food Integrity, Institute of Tropical Agriculture and Food Security (ITAFoS), Universiti Putra Malaysia, UPM Serdang, Selangor, Malaysia; ^3^Department of Environmental Engineering and Green Technology, Malaysia-Japan International Institute of Technology, Universiti Teknologi Malaysia, Kuala Lumpur, Malaysia; ^4^Infection and Immunity Program, Department of Microbiology, Biomedicine Discovery Institute, Monash University, Clayton, VIC, Australia

**Keywords:** MOI, one-step growth curve, podoviridae, RFLP, SDS-PAGE, stability, siphoviridae

## Abstract

*Vibrio parahaemolyticus* is a foodborne pathogen that is frequently isolated from a variety of seafood. To control this pathogenic *Vibrio* spp., the implementation of bacteriophages in aquaculture and food industries have shown a promising alternative to antibiotics. In this study, six bacteriophages isolated from the seafood samples demonstrated a narrow host range specificity that infecting only the *V. parahaemolyticus* strains. Morphological analysis revealed that bacteriophages Vp33, Vp22, Vp21, and Vp02 belong to the Podoviridae family, while bacteriophages Vp08 and Vp11 were categorized into the Siphoviridae family. All bacteriophages were composed of DNA genome and showed distinctive restriction fragment length polymorphism. The optimal MOI for bacteriophage propagation was determined to be 0.001 to 1. One-step growth curve revealed that the latent period ranged from 10 to 20 min, and the burst size of bacteriophage was approximately 17 to 51 PFU/cell. The influence of temperature and pH levels on the stability of bacteriophages showed that all bacteriophages were optimally stable over a wide range of temperatures and pH levels. *In vitro* lytic activity of all bacteriophages demonstrated to have a significant effect against *V. parahaemolyticus*. Besides, the application of a bacteriophage cocktail instead of a single bacteriophage suspension was observed to have a better efficiency to control the growth of *V*. *parahaemolyticus*. Results from this study provided a basic understanding of the physiological and biological properties of the isolated bacteriophages before it can be readily used as a biocontrol agent against the growth of *V. parahaemolyticus*.

## Introduction

*Vibrio parahaemolyticus* is a Gram-negative halophilic bacterium, which occurs naturally in marine environments. It is recognized as a leading foodborne pathogen that is frequently isolated from a variety of seafood (Wang et al., 2015). Consumption of food contaminated with *V. parahaemolyticus* can cause gastroenteritis, which is often displayed through bouts of abdominal pain, diarrhea, fever, and nausea. An open wound in the skin which comes in contact with *V. parahaemolyticus* can also result in wound infection. In rare instances, *V. parahaemolyticus* can cause life-threatening conditions like rapid necrosis of subcutaneous tissue and septicemia (Tena et al., 2010; Ahmad et al., 2013; Zhang and Orth, 2013). However, not all *V. parahaemolyticus* strains are pathogenic and capable of causing illness in humans (Elmahdi et al., 2016). The presence of thermostable direct hemolysin (TDH) and the TDH-related hemolysin (TRH) has been identified as well-known pathogenic factors in *V. parahaemolyticus* which cause vibriosis. Both *tdh* and *trh* genes are therefore widely used as a virulence marker in many studies for the detection of pathogenic *V. parahaemolyticus* strains (Chen et al., 2016; Kang et al., 2017; Paria et al., 2019). However, infection caused by *V. parahaemolyticus* strains without the virulence *tdh* and *trh* genes have been reported in numerous studies (Bhoopong et al., 2007; Jones et al., 2012; Li et al., 2014; Pazhana et al., 2014). The pathogenesis of *V. parahaemolyticus* is therefore still open to question and has not been fully elucidated.

Bacteriophages or phages are bacterial viruses that infect bacterial cells only. Bacteriophages can be found in ubiquitous environments and are recognized as the most abundant organisms with 10^31^ bacteriophage particles on the earth’s surface (Comeau et al., 2008; Keen, 2015). Bacteriophages are typically highly host-specific, which targets only specific species or even strains (Hagens and Loessner, 2007). Besides, high specificity levels of bacteriophages could minimize the disturbance of commensal bacteria (Viazis et al., 2011). The International Committee on Taxonomy of Viruses (ICTV) is responsible for the classification and nomenclature of viruses based on their morphological, genomic and proteomic characteristics (Adams et al., 2017). Tailed bacteriophages are grouped into the order of Caudovirales which consist of three families *Myoviridae*, *Podoviridae*, and *Siphoviridae*. Today, the order of Caudovirales includes six subfamilies, 80 genera, and 441 species (Krupovic et al., 2016). In 2018 and 2019, an update from the ICTV has proposed the creation of a new order (*Tubulavirales*), 10 new families (*Autographiviridae*, *Chaseviridae*, *Demerecviridae*, *Drexlerviridae*, *Finnlakeviridae*, *Halspiviridae*, *Herelleviridae*, *Ovaliviridae*, *Plectroviridae*, and *Thaspiviridae*), 22 subfamilies, 424 genera, and 964 species (Adriaenssens et al., 2020).

Bacteriophages can be grouped into individual families based on their distinct morphological structure, as viewed through electron microscopy (Schramlová et al., 2010). Electron microscopy such as transmission electron microscopy (TEM) is typically used at the initial stage for rapid identification and characterization of viruses. Genetic materials of viruses can be either DNA or RNA, and the structure of the nucleic acid may be either single-stranded or double-stranded. Restriction enzyme cleaves the DNA of bacteriophages into distinct polymorphism, which is known as restriction fragment length polymorphism (RFLP). The use of RFLP analysis can facilitate the study of genetic diversity and differentiate between individual bacteriophages. On the other hand, protein profiles of bacteriophages can be analyzed by sodium dodecyl sulfate-polyacrylamide gel electrophoresis (SDS-PAGE). SDS-PAGE analysis of phage structural proteins will reveal the molecular weight of phage proteins and help to determine the presence of phage structural proteins.

It is noteworthy that the discovery of bacteriophages occurred much earlier than antibiotics. The use of bacteriophage preparations in human trials have also been conducted for almost a century in Eastern Europe (Lin et al., 2017). Nevertheless, the development of a broad-spectrum of antibiotics later received more attention than the use of bacteriophages. Today, phage therapy involves the use of bacteriophages in clinical trials that have gained new attention due to the growing number of infections caused by multidrug-resistance bacteria (Rohde et al., 2018). Phage therapy against several bacterial pathogens in animal models have also been tested out and showed effective results in many studies (Biswas et al., 2002; Wang et al., 2006; Watanabe et al., 2007; Jun et al., 2014; Kalatzis et al., 2018).

The application of bacteriophages is not only limited to clinical trials to fight against bacterial infections. It can be used in livestock protection to reduce the mortality rate of animals as well as enhance the food safety levels by controlling the growth of foodborne pathogens. Globally, efforts to control *Vibrio* spp. with the application of bacteriophages in aquaculture settings have been reported by researchers. For example, Wang et al. (2017) demonstrated that the use of bacteriophage therapy in controlling *V. harveyi* within greenlip abalone. Le et al. (2020b) showed that the application of lytic bacteriophages in the reduction of oyster larvae mortality rate caused by *V. alginolyticus*. Le et al. (2020a) demonstrated that the effectiveness of bacteriophages treatment in the decontamination of *Vibrio* spp. in commercially produced microalgae that used as the primary food source for oyster larvae during hatchery culture. In the future, it is believed that the use of bacteriophages could be a promising alternative to antibiotics for fighting against bacterial pathogens.

In this study, the objective was to isolate *V. parahaemolyticus* bacteriophages from the seafood samples and the isolated bacteriophages were characterized based on host specificity, morphological, genomic character, protein profile, and temperature and pH stability. The *in vitro* lytic activity of single bacteriophage suspensions as well as the bacteriophage cocktails were also evaluated to determine their efficiency against the growth of *V. parahaemolyticus.*

## Materials and Methods

### Bacterial Strains

Six *V. parahaemolyticus* strains which consisted of 4 clinical isolates and 2 food source isolates (non-*tdh* and/or *trh*) were used as hosts for the isolation of bacteriophages (Table 1). PSU5333, PSU5322, and PSU4211 *V. parahaemolyticus* strains were kindly provided by Professor Dr. Varaporn Vuddhakul from the Prince of Songkla University, Thailand. *V. parahaemolyticus* ATCC 17802 was purchased from the American Type Culture Collection (ATCC), United States. Both VP8 and VP11 isolates were obtained from the laboratory stock strain at Food Safety Laboratory, Universiti Putra Malaysia. All bacterial strains used were confirmed by PCR amplification of species-specific *toxR* gene and pathogenicity *tdh* and *trh* genes with specific primers as mentioned in the previous study (Tan et al., 2017) and the amplified sequences were further confirmed by DNA sequencing.

**TABLE 1:** *V. parahaemolyticus* strains used for bacteriophages isolation.

**Isolate ID**	**Source**	**Pathogenic factor**	
		***tdh***	***trh***
PSU5333	Clinical	+	−
PSU5322	Clinical	+	+
PSU4211	Clinical (O3:K6 serotype)	+	+
ATCC 17802	ATCC	−	+
VP8	Fish	−	−
VP11	Fish	−	−

### Sample Preparation

A total of 30 seafood samples consisting of 10 samples for each blood clam, shrimp, and surf clam were purchased from different wet markets in Selangor, Malaysia. Ten grams of each sample was then weighed and transferred into 10 mL of salt of magnesium (SM) buffer (100 mM NaCl, 8 mM MgSO_4_⋅7H_2_O, 50 mM pH 7.5 Tris-HCl and 0.01% gelatin [Merck, Germany]) in a 50 mL sterile centrifuge tube. The mixture was shaken at 150 rpm for 15 min in an orbital shaker. Large particles were pelleted in the tube via centrifugation at 10,000 × *g* for 5 min. The supernatant was filtered through a 0.2 μM pore size syringe filter (Pall, United States). A 200 μL of log-phase *V. parahaemolyticus* host culture (OD_600__*nm*_ = 0.4–0.6) was added into the 5 mL of filtrate and incubated at 37°C, 150 rpm in an orbital shaker overnight. After incubation, the mixture was centrifuged at 10,000 × *g* for 5 min to pelletize the bacterial cells and the supernatant was filtered with a 0.2 μM pore size syringe filter (Pall, United States).

### Detection of the Presence of Bacteriophages

Ten-fold serial dilutions (through 10^–8^) of the filtrate was prepared in SM buffer. Each of these different dilutions was screened for the presence of bacteriophage by spotting 10 μL of the filtrate on a double-layer agar plate with a bacterial host (Beck et al., 2009). The plate was allowed to dry for at least 15 min and the plate was incubated in an inverted position at 37°C overnight. Lysis zone was observed after the incubation. Filtrate from the highest dilution which showed a clear lysis zone on the double-layer agar plate was selected for isolation, propagation, and purification.

### Isolation of Bacteriophage

Isolation of bacteriophages was carried out according to the double agar overlay plaque assay (Kropinski et al., 2009). A 100 μL of each dilution filtrate and 100 μL of the log-phase bacteria culture was aliquot into a 3 mL molten soft agar, which constituted of tryptic soy broth (TSB) (Merck, Germany) and 0.6% bacteriological agar (Merck, Germany), which was stored at 45°C. The molten soft agar was mixed gently and poured evenly onto tryptic soy agar (TSA) (Merck, Germany) base plate. The plate was allowed to solidify for at least 15 min and incubated in an inverted position at 37°C overnight. After incubation, a single colony plaque with clear lysis appearance from the highest dilution was picked using a sterile inoculation loop and transferred it into a 5 mL SM buffer with 200 μL log-phase bacteria culture. The mixture was incubated at 37°C, 150 rpm in an orbital shaker overnight. After incubation, the sample was centrifuged at 10,000 × *g* for 5 min, and the supernatant was filtered with a 0.2 μM pore size syringe filter.

### Propagation and Precipitation of Bacteriophage

Bacteriophage was amplified according to the phage propagation via liquid lysate as described by Bonilla et al. (2016) but with some modifications. Briefly, 200 mL of TSB supplemented with 0.001 M CaCl_2_ and MgCl_2_ was prepared in a 250 mL centrifuge bottle and spiked with 0.1 volumes of overnight bacteria host. A 200 μL of bacteriophage lysate (>10^8^ PFU/mL) was then added into the centrifuge bottle and incubated at 37°C, 150 rpm in an orbital shaker until the lysate cleared. After incubation, the sample was centrifuged at 4°C and 4,000 × *g* for 20 min. The supernatant was filtered with a 0.2 μM pore size syringe filter into a sterile centrifuge bottle. The filtrate was precipitated by adding 10% (w/v) polyethylene glycol (PEG) 8000 (Sigma-Aldrich, United States) and kept undisturbed at 4°C for 24 h. After 24 h, the sample was centrifuged at 4°C and 4,000 × *g* for 20 min. The supernatant was removed, and the pellet was suspended in a 10 mL of SM buffer.

### Purification of Bacteriophage

Bacteriophage suspension was purified using caesium chloride (CsCl) (Santa Cruz Biotechnology, United States) with a density gradient according to the method described in the T7 Select^®^ System Manual TB178 (Novagen, United States). A 5 mL of bacteriophage suspension was aliquot slowly onto the uppermost layer and the sample was subjected to ultracentrifugation at 210,000 × *g* and 4°C for 1 h in a Sorvall^TM^ WX ultracentrifuge with a swinging bucket TH-641 Rotor (Thermo Scientific, United States). After centrifugation, a sharp and turbid band containing the purified bacteriophage particles was collected carefully with a Pasteur pipet. The purified bacteriophage was subjected to dialysis to remove CsCl with an Amicon^®^ Ultra-15 100K filter device (Merck, Germany) and SM buffer (Bonilla et al., 2016). After dialysis, the purified bacteriophage was assayed to determine the bacteriophage titer. The purified bacteriophage stock sample was stored at 4°C until further analysis.

### Host Range Specificity

A total of 134 bacteria strains including 126 *V. parahaemolyticus* strains and 8 non-*V. parahaemolyticus* strains were used to study the host range of the isolated bacteriophages. Non-*V. parahaemolyticus* strains including of *V. alginolyticus*, *V. cholerae*, *V. vulnificus*, *Escherichia coli*, *Listeria monocytogenes*, *Salmonella enterica* serovar Typhimurium, *Salmonella enterica* serovar Enteritidis, and *Staphylococcus aureus*. Host range specificity was determined by spotting 20 μL of each bacteriophage (10^5^ PFU/mL) on a double-layer agar plate seeded with a bacteria strain (Viazis et al., 2011). The agar plate with bacteriophage inoculum was allowed to dry for at least 15 min before being incubated at 37°C overnight. Lysis zones were observed on the plate after the incubation.

### Transmission Electron Microscopy (TEM)

Bacteriophage was visualized by a transmission electron microscope (TEM) as described by Kim et al. (2019) with some modifications. A carbon-coated copper grid was placed onto a drop of purified bacteriophage (10^10^ PFU/mL) for 2 min. The grid with the sample was negatively stained first with 2% (w/v) uranyl acetate for 2 min, followed by sterile water for 1 min. The excess sample was removed with a filter paper, and the grid was allowed to air dry for 10 min. The morphology of the bacteriophage was then visualized with a JEM-2100 200-kV transmission electron microscope (JEOL, Japan).

### Bacteriophage Genome Analysis

Extraction of nucleic acids of bacteriophage was performed according to the protocol provided with the Wizard DNA Clean-Up Kit (Promega, United States). For determination of the type of nucleic acids, 2 μL of extracted bacteriophage nucleic acid was treated separately with 2 μL of DNase I (1 U/μL) and RNase A (10 mg/mL) (Thermo Scientific, United States) for 30 min at 37°C (Topka et al., 2019). The treated sample was electrophoresed through 0.7% (w/v) agarose gel at 100 V for 30 min. The agarose gel was stained with ethidium bromide (0.5 μg/ml) (Merck, Germany) and visualized with Syngene GeneGenius BioImaging system, together with GeneSnap software (version 7.12.01) (Syngene, Cambridge, United Kingdom).

### Restriction Fragment Length Polymorphism (RFLP)

Genomic DNA (1 μg/μL) of each bacteriophage was digested individually with *Eco*RI, *Hae*III, *Hin*dIII, and *Sal*I restriction enzymes (Promega, United States) according to the manufacturer’s protocols and incubated at 37°C for 1 h. The digested product was electrophoresed and visualized as mentioned in the above section.

### SDS-PAGE Analysis

Protein profiles of bacteriophage were analyzed by one-dimensional SDS-PAGE. Each purified bacteriophage (10^10^ PFU/mL) sample was first solubilized by mixing an equal volume of 2× sample buffer solution (0.125 M Tris-HCl, 4% w/v SDS, 20% v/v glycerol, 0.01% w/v bromophenol blue, 10% v/v 2-mercaptoethanol) and boiled at 95°C for 10 min (Guan et al., 2004). The solubilized sample was loaded into 5–15% Bullet PAGE One Precast Gel (Nacalai Tesque, Japan) and electrophoresed in 1× running buffer solution (0.25 mol/L Tris, 1.92 mol/L Glycine, and 10 g/L SDS) at 200 V for 30 min. The gel was stained with CBB Stain One Super (Nacalai Tesque, Japan) for 30 min and de-stained several times with deionized water.

### Multiplicity of Infection (MOI)

The multiplicity of infection (MOI) of the bacteriophage was tested according to Liu et al. (2013) with some modifications. The log-phase bacteria host was first prepared and mixed with bacteriophage suspension at different ratios (0.0001, 0.001, 0.01, 0.1, 1, 10, and 100 PFU/CFU). After incubation at 37°C for 3.5 h, the sample was centrifuged at 10,000 × *g* for 5 min. The supernatant was filtered with a 0.2 μM pore size syringe filter and enumerated to determine the bacteriophage titer. Bacteria-free suspension and bacteriophage-free suspension was included in all the experiments as a control sample. An MOI ratio with the highest bacteriophage titer was considered as the optimal MOI.

### One-Step Growth Curve

One-step growth curve of each bacteriophage was studied according to the protocol described by Hong et al. (2014). Briefly, bacteriophage suspension was mixed with 1 mL of log-phase bacteria host culture at the optimal MOI ratio. The mixture was incubated at 37°C and 150 rpm for 10 min to allow the adsorption of bacteriophages to the host cells before centrifuging at 10,000 × *g* for 30 s. The pellet was suspended in 10 mL of TSB and the suspension was incubated at 37°C and 150 rpm. A 100 μL of sample was withdrawn at 10 min intervals up to a total period of 1 h and enumerated by double agar overlay plaque assay in duplicate. The latent period and burst size of the bacteriophage was evaluated from the one-step growth curve.

### Temperature and pH Stability

Temperature and pH stability were tested according to the method described by Thung et al. (2017) with some modifications. The temperature stability of bacteriophage suspension in the SM buffer was tested at different temperatures (−20, 25, 37, 50, 60, and 70°C) and incubated for 2 h. For pH stability, bacteriophage suspension was incubated at different pH ranges (pH 2, 3, 5, 7, 9, and 11) for 2 h. Bacteriophage suspension kept at 4°C in the SM buffer (pH7.5) was used as a control in this study. After incubation, the titer of bacteriophage was determined by double agar overlay plaque assay and the survival rate was calculated by dividing the bacteriophage titer after treatment with the initial bacteriophage concentration in the control sample (Liu et al., 2020).

### *In vitro* Lytic Activity

*In vitro* lytic activity was determined individually for each bacteriophage (Vp33, Vp22, Vp21, Vp02, Vp08, and Vp11) against its respective bacterial host strain. Two bacteriophage cocktails (cocktails A and B) were also prepared and tested for their synergistic effect (Table 2). Bacteriophage cocktails were selected based on the host range specificity results in the section “Host Range Specificity.” Cocktails A and B were prepared for targeting *V. parahaemolyticus* clinical strains and environmental strains, respectively. A 500 μL of log-phase *V. parahaemolyticus* host culture was mixed with an equal volume of bacteriophage lysate (∼1 × 10^6^ PFU/mL). The mixture was incubated immediately at 37°C. A 100 μL sample of the mixture was withdrawn at 0, 2, 4, 6, and 24 h, to determine the concentration of bacteria through spread plating onto a CHROMagar^TM^ *Vibrio* (CV) plate. The plate was allowed to dry for at least 5 min before inverting the plate for incubation.

**TABLE 2:** Bacteriophage cocktail preparations.

**Bacteriophage cocktails**	**Combination of bacteriophages**	**Against combination of bacterial host strains**
Cocktail A	Vp33, Vp22, Vp21, and Vp02	PSU5333, PSU5322, PSU4211, and ATCC 17802
Cocktail B	Vp08 and Vp11	VP8 and VP11

## Results

### Screening of Bacteriophage

A total of six bacteriophages displayed an ability to lyse *V. parahaemolyticus* host strains and caused lysis zones on the double-layer agar plate was discovered in the blood clam, shrimp, and surf clam samples. Out of 10 shrimp samples, four samples were found to have the presence of *V*. *parahaemolyticus* bacteriophages. Only one sample of blood clam and surf clam was detected with the presence of *V*. *parahaemolyticus* bacteriophages.

### Host Range Specificity

Purified bacteriophages were tested for their host range specificity. Lysis zones were observed on 61 out of 126 (48.4%) of the double-layer agar plate seeded with *V. parahaemolyticus* strains (Table 3). No lysis zones were observed on all non-*V. parahaemolyticus* (8/8) bacteria lawn agar plates. Bacteriophages Vp33, Vp22, Vp21, and Vp02, were found to be able to cause lysis zones on 18.3% (23/126) of *V. parahaemolyticus* strains agar plates, which included four clinical strains of *V. parahaemolyticus*. On the other hand, Vp08 and Vp11 were found to be able to cause lysis zones on 34.9% (44/126) of the environmental *V. parahaemolyticus* strains.

**TABLE 3:** Host range specificity on different *V. parahaemolyticus* strains.

**Bacteria strain**	**Isolate ID**	**Number of Isolate**	**Bacteriophage**						**Source**
			**Vp33**	**Vp22**	**Vp21**	**Vp02**	**Vp08**	**Vp11**	
*Vibrio parahaemolyticus*	PSU5333, PSU5322, and PSU4211	3	+	+	+	+	–	–	PSU
	ATCC 17802	1	+	+	+	+	–	–	ATCC
	BC29, SC12, SC24, SH2, SQ13, and SQ28	6	+	+	+	+	+	+	FSQL
	BC13, BC15, BC32, SC16, SC19, SC27, SH11, SH13, SH23, SH27, SH8, SQ20, and SQ22	13	+	+	+	+	−	−	FSQL
	VP8, VP11, BC1, BC6, BC7, BC8, BC9, BC10, BC11, BC18, SC17, SC22, SC25, SC28, SC29, SC1, SC5, SC7, SC8, SC11, SH1, SH10, SH12, SH26, SH29, SQ1, SQ11, SQ12, SQ14, SQ18, SQ19, SQ21, SQ23, SQ24, SQ25, SQ26, SQ27, and SQ3	38	−	−	−	−	+	+	FSQL
	BC12, BC16, BC17, BC19, BC2, BC20, BC21, BC22, BC23, BC24, BC25, BC26, BC27, BC28, BC3, BC30, BC31, BC4, BC4, BC5, SC10, SC13, SC14, SC15, SC18, SC2, SC20, SC21, SC23, SC26, SC3, SC4, SC6, SC7, SC9, SH14, SH15, SH16, SH17, SH18, SH19, SH20, SH21, SH22, SH24, SH25, SH28, SH3, SH30, SH31, SH4, SH5, SH6, SH9, SQ10, SQ15, SQ16, SQ17, SQ2, SQ4, SQ5, SQ6, SQ7, SQ8, and SQ9	65	−	−	−	−	−	−	FSQL

*+ Bacteriophage isolated from the sample showed lysis spot.*

*− No bacteriophage isolated from the sample.*

*ATCC, Clinical isolate from American Type Culture Collection; FSQL, Food isolates from Food Safety and Quality Laboratory, Universiti Putra Malaysia; PSU, Clinical isolate from Prince of Songkla University, Thailand.*

### Transmission Electron Microscopy (TEM)

**FIGURE 1:**
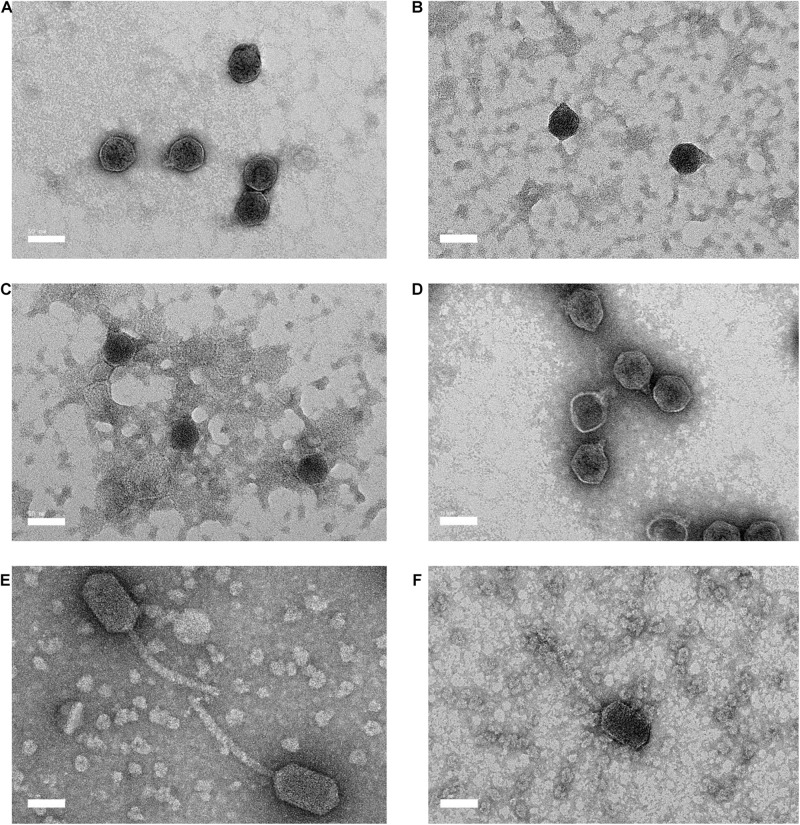
Electron micrography of negative stained *Vibrio parahaemolyticus* bacteriophages and the bar represents 50 nm. **(A)** Vp33 (head diameter: 44.70 ± 2.30 nm; tail diameter: 9.22 ± 0.92 nm); **(B)** Vp22 (head diameter: 41.28 ± 1.83 nm; tail diameter: 8.72 ± 2.29 nm); **(C)** Vp21 (head diameter: 42.63 ± 2.07 nm; tail diameter: 9.86 ± 1.15 nm); **(D)** Vp02 (head diameter: 50.00 ± 3.21 nm; tail diameter: 12.44 ± 2.30 nm); **(E)** Vp08 (head diameter: 91.78 ± 4.11 nm × 49.54 ± 2.05 nm; tail diameter: 142.24 ± 13.47 nm) **(F)** Vp11 (head diameter: 79.00 ± 5.94 nm × 44.98 ± 1.60 nm; tail diameter: 133.79 ± 15.98 nm).

Electron microscopy revealed that the morphological characteristics of bacteriophages Vp33, Vp22, Vp21, and Vp02 were resembled *Podoviridae* family, and bacteriophages Vp08 and Vp11 were resembled *Siphoviridae* family (Figures 1A–F). Bacteriophages Vp33, Vp22, Vp21, and Vp02 were characterized with a hexagonal head diameter between 41.28 ± 1.83 to 50.00 ± 3.21 nm and a short stumpy non-contractile tail diameter between 8.72 ± 2.29 to 12.44 ± 2.30 nm. Bacteriophages Vp08 and Vp11 were exhibited with an elongated hexagonal head diameter between 79.00 ± 5.94 × 44.98 ± 1.60 nm and 91.78 ± 4.11 × 49.54 ± 2.05 nm and a long flexible non-contractile tail diameter between 133.79 ± 15.98 nm and 142.24 ± 13.47 nm.

### Bacteriophage Genome Analysis

From the determination of the nucleic acids, all the bacteriophage nucleic acids were digested by DNase I (1 U/μL) after 30 min incubation at 37°C. None of the bacteriophage nucleic acids was digested by RNase A (10 mg/mL) after 30 min of incubation at 37°C.

### Restriction Fragment Length Polymorphism (RFLP)

**FIGURE 2:**
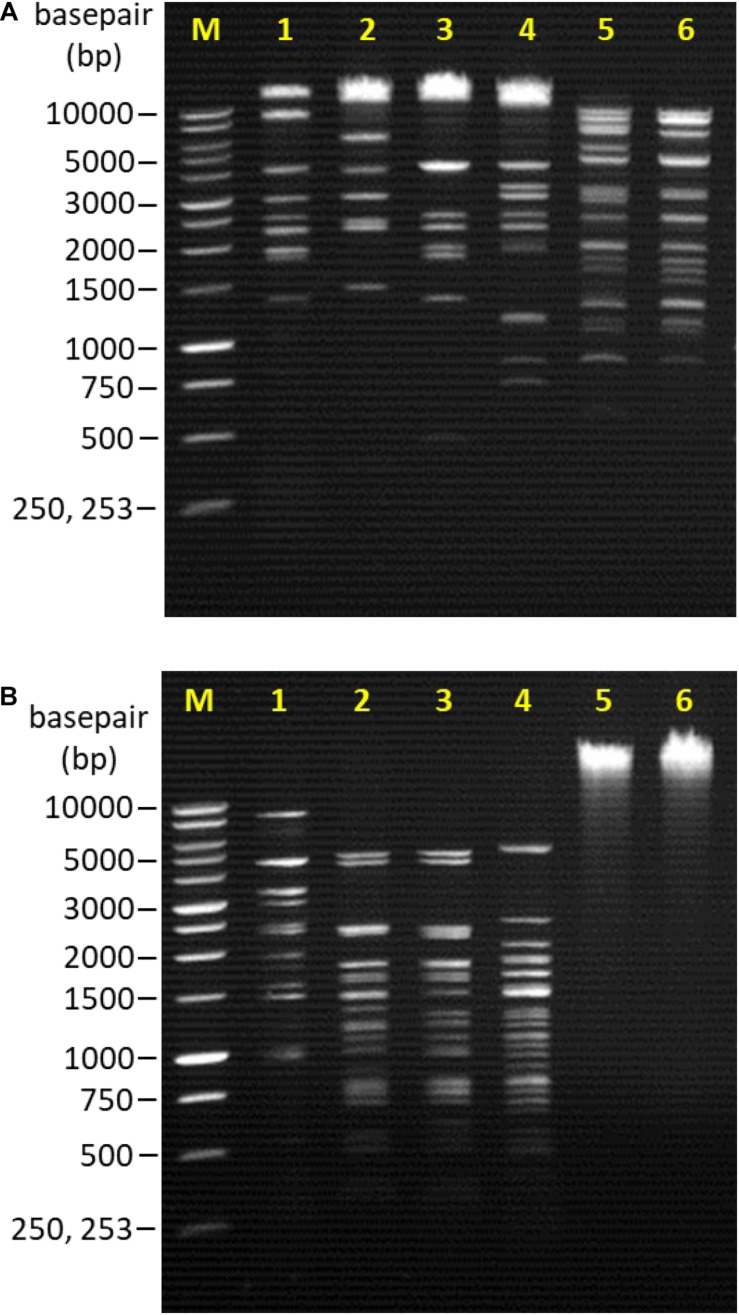
Lane M: 1 kb DNA ladder (Promega, United States); Lane 1: Vp33; Lane 2: Vp22; Lane 3: Vp21; Lane 4: Vp02; Lane 5: Vp08; Lane 6: Vp11. **(A)** RFLP patterns of bacteriophages genomic DNA digested with *Hin*dIII. **(B)** RFLP patterns of bacteriophages genomic DNA digested with *Hae*III.

From the restriction endonuclease digestion, the genomic DNA of all bacteriophages remained intact and unable to be digested by the *Eco*RI and *Sal*I restriction enzymes. However, the genomic DNA of each bacteriophage treated with *Hin*dIII restriction enzyme produced a distinctive RFLP (Figure 2A). *Hae*III restriction digestion profiles were also obtained for all bacteriophages except Vp08 and Vp11 (Figure 2B).

### SDS-PAGE Analysis

**FIGURE 3:**
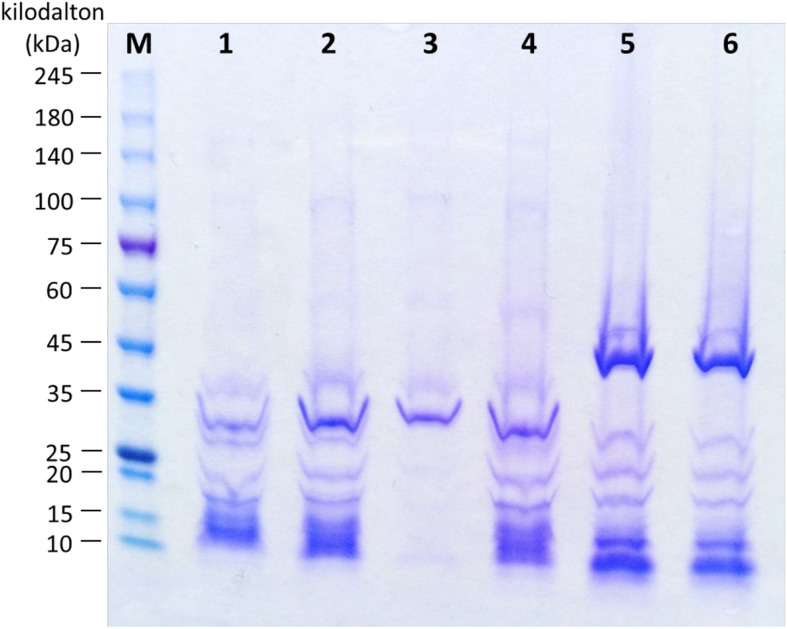
Protein profiles of bacteriophages. Lane M: ExcelBand^TM^ High Range Protein Marker, PM2610 (SMOBIO, Taiwan); Lane 1: Vp33; Lane 2: Vp22; Lane 3: Vp21; Lane 4: Vp02; Lane 5: Vp08; Lane 6: Vp11.

Structural proteins of bacteriophages were analyzed by SDS-PAGE. Protein profiles of bacteriophage Vp33, Vp22, Vp21, and Vp02 demonstrated a major protein band at around 31 kDa, while bacteriophages Vp08 and Vp11 exhibited a major protein band at around 40 kDa (Figure 3). Bacteriophages Vp33, Vp22, and Vp02 were observed with similar protein profiles of 8 clear protein bands at around 36, 31, 26, 20, 17, 15, 12, and 10 kDa. Bacteriophages Vp08 and Vp11 exhibited similar patterns of protein profiles with seven clear protein bands at around 48, 40, 26, 20, 17, 10, and 5 kDa. Bacteriophage Vp21 only showed two clear protein bands at around 35 and 32 kDa.

### Multiplicity of Infection (MOI)

At an MOI of 1, bacteriophages Vp33, Vp08, and Vp11 exhibited the highest bacteriophage titer of 8.34 ± 0.08, 10.05 ± 0.07, and 9.47 ± 0.15 log PFU/mL, respectively, after 3.5 h incubation at 37°C. The MOI ratio of 1 was then determined to be the optimal MOI for the bacteriophages Vp33, Vp08, and Vp11. Bacteriophage Vp22 was observed with an optimal MOI of 0.001 from the highest bacteriophage titer of 9.52 ± 0.06 log PFU/mL, while bacteriophage Vp02 was found with the optimal MOI of 0.1 from the highest bacteriophage titer of 10.02 ± 0.11 log PFU/mL. Lastly, the optimal MOI of bacteriophage Vp21 was determined to be 0.01 to 0.001 with the highest bacteriophage titer of 8.22 ± 0.01 and 8.22 ± 0.03 log PFU/mL.

### One-Step Growth Curve

**FIGURE 4:**
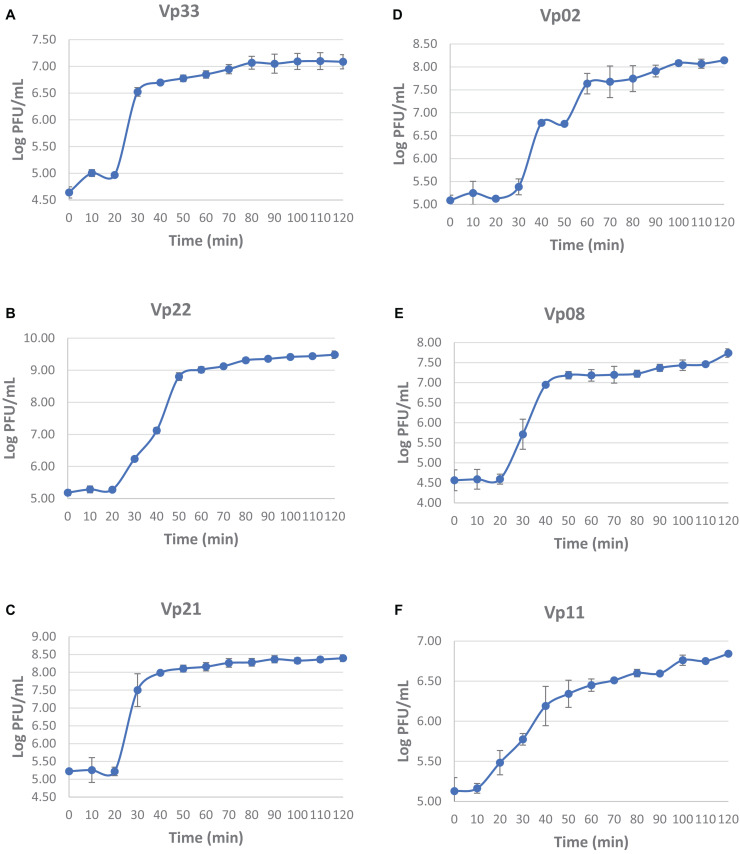
One-step growth curve of bacteriophages. **(A)** Vp33; **(B)** Vp22; **(C)** Vp21; **(D)** Vp02; **(E)** Vp08; **(F)** Vp11.

From the one-step growth curve, all the bacteriophages were observed with a latent period of 20 min except bacteriophage Vp11 showed a faster latent period of 10 min in comparison to the others (Figures 4A–F). The average burst size of bacteriophages Vp33, Vp22, Vp21, Vp02, Vp08, and Vp11 estimated from the one-step growth curve were 22, 51, 34, 26, 37, and 17 plaque-forming units (PFU) per cell, respectively.

### Temperature and pH Stability

All bacteriophages were observed to remain relatively stable at temperatures −20, 25, 37, and 50°C, with survival rate ranged from 93.13 to 100.00% (Table 4). At a temperature of 60°C, a low survival rate of 31.16 and 49.96% was observed in bacteriophages Vp08 and Vp11, respectively. At a temperature of 70°C, all bacteriophages were found inactivated and loss of infectivity. For pH stability, all bacteriophages were observed to be able to withstand a wide range of pH levels from 5 to 11 and found with a percentage of survival of 95.82 to 99.92% (Table 5). Besides, the majority of bacteriophages were found to be resistant at pH3 except that bacteriophages Vp21 and Vp22 were observed with a low survival rate of 47.15 and 52.64%, respectively. At pH2, none of the bacteriophages was survived and recovered after a 2 h period of incubation.

**TABLE 4:** Temperature stability of bacteriophages.

**Temperature**	**Percentage of survival (%)**					
	**Vp33**	**Vp22**	**Vp21**	**Vp02**	**Vp08**	**Vp11**
−20°C	94.58 ± 0.85	96.09 ± 0.11	96.39 ± 2.02	93.13 ± 1.56	99.63 ± 1.21	97.67 ± 1.22
25°C	96.87 ± 0.56	95.61 ± 0.38	100.00 ± 0.07	93.89 ± 1.46	99.29 ± 0.06	99.93 ± 0.17
37°C	97.98 ± 0.55	97.90 ± 0.75	97.69 ± 0.92	95.60 ± 1.42	99.92 ± 0.50	99.38 ± 0.01
50°C	97.76 ± 0.49	97.62 ± 0.05	99.27 ± 0.56	95.69 ± 0.51	99.33 ± 0.75	98.79 ± 0.56
60°C	93.30 ± 0.82	95.54 ± 0.72	95.30 ± 0.93	91.15 ± 1.25	31.16 ± 4.58	49.96 ± 2.60
70°C	0.00	0.00	0.00	0.00	0.00	0.00

**TABLE 5:** pH stability of bacteriophages.

**pH**	**Percentage of survival (%)**					
	**Vp33**	**Vp22**	**Vp21**	**Vp02**	**Vp08**	**Vp11**
2	0.00	0.00	0.00	0.00	0.00	0.00
3	98.28 ± 1.50	52.64 ± 7.31	47.15 ± 1.59	94.30 ± 1.15	96.67 ± 1.29	94.94 ± 0.81
5	99.54 ± 0.13	100.05 ± 0.57	99.36 ± 0.45	99.04 ± 0.67	97.52 ± 0.21	98.76 ± 0.87
7	100.79 ± 0.93	98.68 ± 0.49	99.85 ± 0.12	97.58 ± 0.74	98.84 ± 0.38	102.21 ± 0.22
9	99.92 ± 0.02	100.39 ± 0.25	99.82 ± 0.33	99.47 ± 2.79	95.82 ± 0.59	100.13 ± 1.21
11	100.48 ± 0.30	100.57 ± 0.00	98.96 ± 0.96	100.10 ± 0.22	96.86 ± 1.47	99.35 ± 0.53

### *In vitro* Lytic Activity

All bacteriophages, including two bacteriophage cocktail preparations, were demonstrated to have significant (*P* < 0.05) *in vitro* lytic activity after 2 h incubation at 37°C (Table 6). The efficiency of single bacteriophage suspensions as well as the bacteriophage cocktails were also observed to increase significantly over time. The total reduction was reported to be in the range of 2.85 ± 0.07 to 4.60 ± 0.08 log CFU/mL, for individual bacteriophage suspension. Bacteriophage cocktails A and B achieved a better total reduction, with 3.69 ± 0.49 and 4.81 ± 0.14 log CFU/mL, respectively.

**TABLE 6:** Viable count (log CFU/mL) of *V. parahaemolyticus* after 0, 2, 4, 6, and 24 h incubation at 37°C with bacteriophage.

**Sample**	**Time (h)**					**Total Reduction (after 24 h)**
	**0**	**2**	**4**	**6**	**24**	
Vp33	7.08 ± 0.15	6.13 ± 0.09	5.73 ± 0.20	4.30 ± 0.08	3.70 ± 0.34	3.38 ± 0.26
Vp22	7.06 ± 0.22	6.06 ± 0.11	5.41 ± 0.33	5.02 ± 0.17	3.93 ± 0.26	3.20 ± 0.25
Vp21	7.13 ± 0.11	6.19 ± 0.10	5.63 ± 0.07	5.12 ± 0.11	4.07 ± 0.12	2.85 ± 0.07
Vp02	6.77 ± 0.13	5.93 ± 0.09	5.81 ± 0.18	5.13 ± 0.15	3.92 ± 0.11	2.98 ± 0.22
Vp08	7.81 ± 0.18	6.23 ± 0.12	5.52 ± 0.09	4.63 ± 0.28	3.59 ± 0.25	4.22 ± 0.26
Vp11	7.96 ± 0.13	6.33 ± 0.04	5.49 ± 0.28	4.62 ± 0.10	3.36 ± 0.20	4.60 ± 0.08
Cocktail A	7.09 ± 0.06	6.11 ± 0.10	5.78 ± 0.24	4.09 ± 0.07	3.40 ± 0.54	3.69 ± 0.49
Cocktail B	8.01 ± 0.15	6.15 ± 0.21	5.13 ± 0.24	4.33 ± 0.10	3.20 ± 0.07	4.81 ± 0.14

*Total reduction was determined by calculating the reduction achieved between initial (0 h) and final bacteria concentration (24 h). Each data point represents mean result ± standard deviation in duplicates.*

## Discussion

The majority of studies were focused on the isolation of bacteriophages from environmental sources such as lake sediment, poultry farm, seawater, sewage, and soil (Salifu et al., 2013; Shende et al., 2017; Huang et al., 2018; Akhwale et al., 2019; Kim et al., 2019; Tang et al., 2019; Necel et al., 2020; Sevilla-Navarro et al., 2020). Reports of the isolation of these bacteriophages presence on fresh food samples were relatively limited in extent. According to Greer (2005), the successful isolation of bacteriophages from food samples was reliant on the occurrence of the target bacteria which was present in the food samples in relatively high concentrations.

Shrimp samples purchased from the wet markets were found to be the primary source of *V. parahaemolyticus* bacteriophages in this study. Similarly, several studies reported the isolation of *Vibrio* spp. bacteriophages from a similar source such as shrimp aquaculture environments (Alagappan et al., 2010; Stalin and Srinivasan, 2017; Matamp and Bhat, 2020). For instance, Alagappan et al. (2010) reported that five bacteriophages with the ability to infect *V. parahaemolyticus* MTCC-451 strain were isolated from the shrimp pond. Stalin and Srinivasan (2017) reported that bacteriophage VVP1 capable of infecting *V. parahaemolyticus* as well as *V. alginolyticus* strains were isolated from the water suspended sediment samples collected from the shrimp grow-out pond. Matamp and Bhat (2020) also reported that bacteriophages ⌽VP-1 which able to lyse five strains of *V. alginolyticus* and 1 strain of *V. parahaemolyticus* were isolated from the shrimp pond water samples.

Host specificity of bacteriophage is one of the vital aspects which should be considered in a bacteriophage application. The specificity of bacteriophages isolated in this study were found to be of a narrow host range that infected only *V. parahaemolyticus* strains. A narrow host-specific bacteriophage seemed an attractive feature, especially when applying it in the gastrointestinal tract to target specific host bacteria as the narrow host range of the bacteriophage would not affect other endogenous bacteria (Viazis et al., 2011; Drulis-Kawa et al., 2012). Besides, different types of narrow host range bacteriophages can be mixed in a cocktail preparation for a more effective *in vitro* control of pathogenic bacteria (Tanji et al., 2004; Mapes et al., 2016; Bai et al., 2019).

Both the *Podoviridae* and *Siphoviridae* families are classified under the same order of *Caudovirales*. A hexagonal head (41.28 to 50.00 nm) and a short stumpy non-contractile tail (8.72 to 12.44 nm) which resembled the *Podoviridae* family was observed in the negative staining of bacteriophages Vp33, Vp22, Vp21, and Vp02 under TEM. Similarly, Zhang et al. (2018) reported that a *V. parahaemolyticus* bacteriophage vB_VpaS_OMN isolated from the seafood sample belonged to the *Podoviridae* family and was observed to have a 55 nm isometric capsid and a short tail of about 16 nm. On the other hand, the electron micrographs of bacteriophages Vp08 and Vp11 showed an elongated hexagonal head (79.00 × 44.98 to 91.78 × 49.54 nm) and a long flexible non-contractile tail (133.79 to 142.24 nm). These morphological characteristics resemble those of the *Siphoviridae* family. A recent study by Yang et al. (2020) reported a similar finding that two *V. parahaemolyticus* bacteriophages, namely vB_VpS_BA3 and vB_VpS_CA8, isolated from the sewage were *Siphoviridae* bacteriophage with an icosahedral head (70 × 55 nm) and a long non-contractile tail (125 to 130 nm).

Dion et al. (2020) reported that double-stranded DNA (dsDNA) tailed bacteriophages accounted for the majority of bacteriophages reported in the public database. This finding supports with Dion et al. (2020) statement as all bacteriophages in this study encapsulate a dsDNA genome. From the restriction endonuclease digestion, some genetic variation or polymorphisms were observed among bacteriophages. Distinctive restriction digestion profiles between each bacteriophage isolates were observed after the genome of bacteriophages was digested by the *Hin*dIII and *Hae*III restriction enzymes. From the restriction endonuclease digestion profiles, it can be concluded that all bacteriophage isolates analyzed in this study present some variation in their genetic properties.

A prominent protein band at around 31 kDa was identified by SDS-PAGE analysis in all *Podoviridae* bacteriophages, Vp33, Vp22, Vp21, and Vp02. In contrast, bacteriophages Vp08 and Vp11 showed a prominent protein band at around 40 kDa. This prominent protein was assumed to be the major capsid protein of bacteriophage. In other studies, Drulis-Kawa et al. (2012) revealed a 39 kDa major capsid protein in the *Klebsiella pneumoniae* podovirus bacteriophage, and this protein structure was found highly homologous (78%) to the 37.8 kDa *Vibrio* bacteriophage VP93 capsid protein. Baudoux et al. (2012) revealed that a *Vibrio* siphovirus bacteriophage SIO-2 was identified with the presence of a major capsid protein with a molecular mass of 29 kDa. Other protein bands may indicate the presence of putative tail fiber protein (32.7 kDa), putative scaffolding protein (30.1 kDa), putative internal virion protein B (20.4 kDa), hypothetical and/or unknown proteins of *Vibrio* bacteriophage (Drulis-Kawa et al., 2012; Hu et al., 2020).

Bacteriophages rely on the chance of meeting with bacteria to infect and kill bacteria (Huff et al., 2006). The MOI refers to the ratio of the virus particles to the host cells. In this study, the optimal MOI for bacteriophage propagation was determined to be 0.001 to 1. Different bacteriophages may infect and kill bacteria differently, thus having different optimal MOIs. For example, Yang et al. (2020) reported that two *V. parahaemolyticus* lytic bacteriophages, vB_VpS_BA3 and vB_VpS_CA8, were tested with optimal MOI of 0.1 from the maximum bacteriophage titer produced. A lytic bacteriophage PhVh6 showing the ability to lyse *V. alginolyticus*, *V. harveyi*, and *V. parahaemolyticus* was tested with an optimal MOI of 1 (Ibrahim et al., 2017).

From the one-step growth curve, the latent period of bacteriophages was estimated to be around 10 to 20 min and the average burst size was anticipated to be approximately 17 to 51 PFU/cell. Previously, vibriophage BA3 and CA8 were estimated as having a latent period of around 20 to 30 min and a burst size of about 100 to 180 PFU/cell (Yang et al., 2020). Another lytic bacteriophage VP06 of *V. parahaemolyticus* was estimated to have a latent period of approximately 30 min and 60 PFU per infected bacterial cell (Wong et al., 2019). Lal et al. (2016) also reported that a *V. parahaemolyticus* bacteriophage VpKK5 exhibited a latent period of 36 min and a burst size of 180 PFU/cell. From the results, bacteriophage isolates in this study demonstrated a shorter latent period but a smaller burst size than other reported vibrio bacteriophages.

Physico-chemical factors such as temperature and pH level can affect the effectiveness of bacteriophage applications against pathogenic bacteria (Ly-Chatain, 2014). For example, higher temperatures may result in irreversible damage or denaturation of the virus particles (Ahmadi et al., 2017). The concentration of hydrogen ions in an acidic solution can cause a decline in bacteriophage concentration due to the aggregation process (Langlet et al., 2007; Jończyk et al., 2011). In the current study, the effect of temperature on bacteriophage stability revealed that all bacteriophages remained relatively stable at temperatures ranging from −20 to 50°C. At 60°C, bacteriophages Vp33, Vp22, Vp21, and Vp02 which belong to the *Podoviridae* family showed continuing stability. In contrast, bacteriophages Vp08 and Vp11 which belong to the *Siphoviridae* family showed a low survival rate at 60°C. Incubation at 70°C showed total inactivation of all bacteriophages. Previous studies reported a similar finding, in which *V. parahaemolyticus* bacteriophage VVP1 was found to be stable at temperatures 4 to 50°C but completely inactive at 60°C (Stalin and Srinivasan, 2017). Investigation of the effect of pH on bacteriophage stability revealed that all bacteriophages sustained well at pH ranges of 5 to 11. At pH3, bacteriophages Vp22 and Vp21 were observed with a low survival percentage. At pH2, none of the bacteriophages survived this highly acidic environment. Such characteristics align with results reported by Jun et al. (2014) in which a bacteriophage pVp-1, showed infectivity for multiple–antibiotic-resistant *V. parahaemolyticus* and *V. vulnificus*, was observed to be stable over a wide range of pH levels of 3 to 11.

Overall, the bacteriophage suspensions achieved an average log reduction of *V. parahaemolyticus* in the range of 2.85 ± 0.07 to 4.81 ± 0.14. Bacteriophage cocktail preparations were observed to have a better *in vitro* lytic activity as compared to a single bacteriophage suspension. However, other attributes such as competition between each *V. parahaemolyticus* strain and production of antagonistic compounds by *V. parahaemolyticus* strains that may exhibit antibacterial activity were excluded in the results and should be considered in the further study. Bacteriophage cocktail A achieved a total reduction rate of 3.69 ± 0.49 log CFU/mL, compared to its individual bacteriophage suspension with a reduction rate between 2.85 ± 0.07 and 3.38 ± 0.26 log CFU/mL. Bacteriophage cocktail B achieved a total reduction rate of 4.81 ± 0.14 log CFU/mL, compared to its individual bacteriophage suspension with a reduction rate between 4.22 ± 0.26 and 4.60 ± 0.08 log CFU/mL. According to these results, the application of a bacteriophage cocktail instead of a single bacteriophage suspension could therefore result in an improved reduction in the growth of *V*. *parahaemolyticus*.

Mateus et al. (2014) stated that the use of bacteriophage cocktails mixed with two or three bacteriophages can increase the efficiency to control and inactivate the growth of *V*. *parahaemolyticus* better than the single bacteriophage suspensions. For example, Mateus et al. (2014) reported that the application of bacteriophage cocktails mixed with the VP-1 and VP-2 bacteriophages achieved a maximum of 4.0 log of *V*. *parahaemolyticus* inactivation after 2 h of incubation, compared to the use of VP-1 and VP-2 bacteriophage alone, with a reduction rate of only 0.8 log. Similarly, Ren et al. (2019) reported that a bacteriophage cocktail which consisted of PVP1 and PVP2 bacteriophages was superior in preventing *V*. *parahaemolyticus* infections in sea cucumbers than the use of single bacteriophages. It was also recently demonstrated that the benefit of bacteriophage cocktails can help to overcome the development of bacteriophage-resistant pathogen strains (Yang et al., 2020). For instance, Kim et al. (2020) demonstrated that a bacteriophage cocktail consists of three types of *V. coralliilyticus*-specific bacteriophages with greater efficiency against multiple-antibiotic-resistant (MAR) and phage-resistant (PR) *V. coralliilyticus* infection in oyster larvae. Yen et al. (2017) reported that a bacteriophage cocktail that targets different receptors on *V. cholerae* would reduce the emergence of bacteriophage-resistant *V. cholerae* isolates. Hence, bacteriophage cocktails consisting of multiple bacteriophage suspensions are more effective in the lytic ability.

## Conclusion

*Vibrio parahaemolyticus* specific bacteriophages can be readily recovered from seafood samples. Bacteriophage Vp33, Vp22, Vp21, Vp02, Vp08, and Vp11 isolated from the seafood samples exhibited narrow host range and lytic activity against *V. parahaemolyticus* strains only. Characterization of bacteriophages in this study facilitates a better understanding of the physiological and biological features of bacteriophages. The results of *in vitro* lytic activity revealed the usability of bacteriophages as biocontrol agents for the control of *V. parahaemolyticus.*

## Data Availability Statement

The raw data supporting the conclusions of this article will be made available by the authors, without undue reservation.

## Author Contributions

NA-M, NJ, TT, and EL provided assistance and guidance throughout the research. YR, HaH, HiH, and SR were the mentor in the research study and assist in manuscript checking. All authors contributed to the article and approved the submitted version.

## Conflict of Interest

The authors declare that the research was conducted in the absence of any commercial or financial relationships that could be construed as a potential conflict of interest.
